# Amidoximes and Oximes: Synthesis, Structure, and Their Key Role as NO Donors

**DOI:** 10.3390/molecules24132470

**Published:** 2019-07-05

**Authors:** Tanya Sahyoun, Axelle Arrault, Raphaël Schneider

**Affiliations:** 1 Laboratoire de Chimie Physique Macromoléculaire, Université de Lorraine, CNRS, LCPM, F-54000 Nancy, France; 2 Laboratoire Réactions et Génie des Procédés, Université de Lorraine, CNRS, LRGP, F-54000 Nancy, France

**Keywords:** amidoxime, oxime, synthesis, isomerism, nitric oxide, oxidation

## Abstract

Nitric oxide (NO) is naturally synthesized in the human body and presents many beneficial biological effects; in particular on the cardiovascular system. Recently; many researchers tried to develop external sources to increase the NO level in the body; for example by using amidoximes and oximes which can be oxidized in vivo and release NO. In this review; the classical methods and most recent advances for the synthesis of both amidoximes and oximes are presented first. The isomers of amidoximes and oximes and their stabilities will also be described; (*Z*)-amidoximes and (*Z*)-oximes being usually the most energetically favorable isomers. This manuscript details also the biomimetic and biological pathways involved in the oxidation of amidoximes and oximes. The key role played by cytochrome P450 or other dihydronicotinamide-adenine dinucleotide phosphate (NADPH)-dependent reductase pathways is demonstrated. Finally, amidoximes and oximes exhibit important effects on the relaxation of both aortic and tracheal rings alongside with other effects as the decrease of the arterial pressure and of the thrombi formation

## 1. Introduction

**Figure 1:**
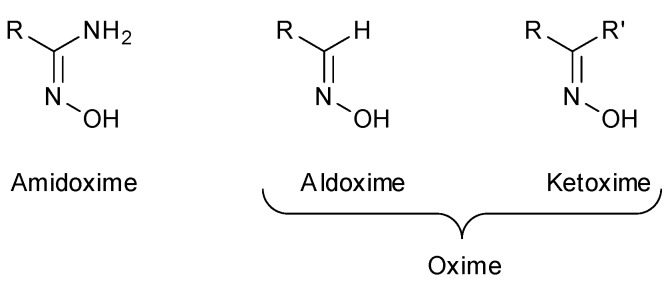
Structures of amidoximes and oximes.

In recent years, oximes and amidoximes (oximes in which one of the substituents is an amino group) (Figure 1) have gained high interest. These compounds are usually easy to synthesize and were studied in many different fields such as coordination [1] or materials chemistry [2,3] but also for their numerous biological activities. Moreover, the amidoxime function is often used as bioisoster of a carboxylic acid, and there are some successful examples of drug candidates exhibiting cardiotonic or antiarthritic properties containing the amidoxime moiety.

**Figure 2:**
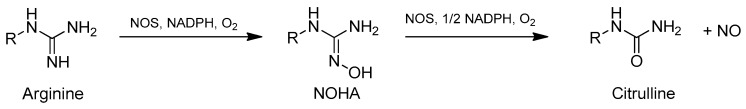
In vivo NO synthesis from arginine.

Oximes and amidoximes have also gained high interest regarding their ability to release nitric oxide (NO). The oxidation of these compounds can be catalyzed by various hemoproteins like cytochrome P450 (CYP450) or horseradish peroxidase (HRP) [4]. The first step of arginine oxidation has been extensively studied since the intermediate product, *N*-hydroxy-l-arginine (NOHA) exhibiting an amidoxime function, is oxidized by NO-synthase (NOS) and other enzymes like CYP450 into NO (Figure 2) [5]. NO is involved in many physiological processes such as neurotransmission, blood pressure regulation, or immunomodulation. Thus, it is important to have external sources of NO specially when NOS presents an abnormal activity as it is the case for patients having diseases like diabetes or hypertension [6,7]. For this reason, exogenous compounds able to be oxidized by different pathways that do not involve NOS are of high interest.

Microsomal oxidation of amidoximes generates the corresponding amides and/or nitriles. It has been demonstrated that these oxidative cleavages of C=N bonds to the corresponding C=O bonds result in a transfer of one oxygen atom (dependent on CYP450) from O_2_ to the substrate, with simultaneous release of NO [8].

**Figure 3:**
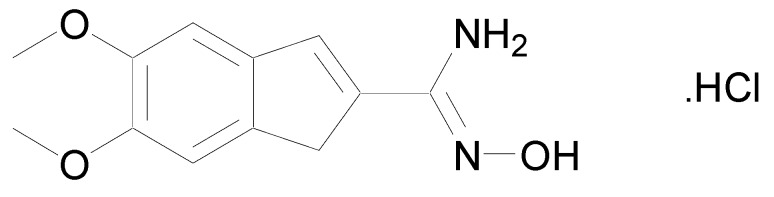
Structure of *N*-hydroxy-5,6-dimethoxy-1*H*-indene-2-carboximidamide.HCl.

In 1986, a patent was deposited for a series of compounds containing an amidoxime function possessing cardiotonic activities and allowing an increase in the heart muscle force [9]. In 1989, a second patent proved that many compounds bearing amidoxime functions can reduce heart failures by increasing the contractile force of the heart muscle [10]. These studies were followed by the work of Shahid et al. who demonstrated that once the cardiotonic agent depicted in Figure 3 injected in isolated cardiac and vascular tissues, positive chronotropic and inotropic effects were observed [11].

The oxidations of oximes and amidoximes were studied in vitro using either chemical reagents like 2-iodobenzoic acid (IBX) or IBX associated to tetraethylammonium bromide (TEAB) [12] or biological ones like microsomes of rats containing CYP450 [5]. Amidoximes are also known to be reduced in vivo into amidines exhibiting important anti-microbial activities against many pathogens such as pneumocystis and protozoan [13,14].

In this review, we focus on the oxidation methods of both amidoximes and oximes developed over the last twenty years, topic that has never been described to date. The first part is devoted to the structure and isomerism of these compounds, which is an important subject only scarcely presented in the literature. Next, the major methods developed for the synthesis of amidoximes and oximes will be reported. Finally, the last part will describe the in vitro oxidation steps of amidoximes and oximes via chemical as well as biological processes. The oxidation mechanisms will also be presented in order to describe the exact pathways of NO release. Finally, the last part will focus on the in vivo oxidations of amidoximes and oximes in rats and rabbits alongside with their effects like the release of NO, the blood pressure regulation and the vasodilatation.

## 2. Isomerism of Amidoximes and Oximes

Amidoximes and oximes have poorly been studied in the literature regarding their isomerism. However, this topic is of high interest for the determination of their structure. These two compounds can exhibit two types of isomers, (i) geometrical diastereoisomers *Z* or *E*, and (ii) constitutional isomers (tautomers). Better knowledge of the relative stability of the tautomeric forms of amidoximes would permit to elucidate some aspects of their pharmacological activity, of their reactivity and of their ability to coordinate with metal centers.

**Figure 4:**
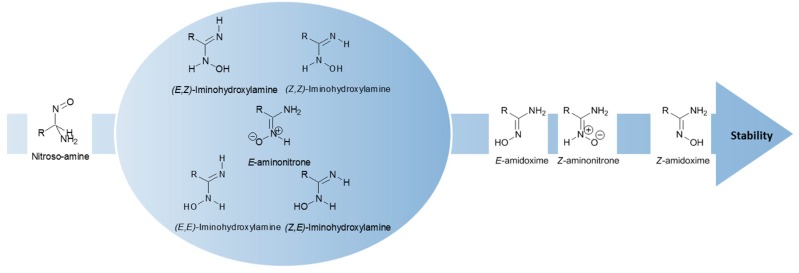
Summary of the stability of amidoxime isomers.

Some studies were devoted to amidoximes in order to clarify their structure. Four main amidoxime tautomers plus their geometrical isomers (*Z/E*) were studied, namely amidoxime, iminohydroxylamine, aminonitrone and nitroso-amine (Figure 4) [15,16]. In 1964, the presence of a zwitterionic form for amidoximes was demonstrated. This zwitterion or aminonitrone was identified via FT-IR by the presence of a medium to strong band at 1690 cm^−1^ associated to the amidoxime C=N stretching at 1656 cm^−1^. The main aminonitrone and amidoxime IR bands are summarized in Table 1 [17].

Recent theoretical and experimental studies show that the most stable and dominant form is the *Z*-amidoxime [15,16,18]. This isomer is more stable than the iminohydroxylamine, the aminonitrone and the nitroso-amine, the latter being the less stable [18]. However, according to the most recent published work on that topic [16], three amidoximes isomers may coexist due to their close relative energies: the *Z*-amidoxime as the most stable form in both protic and aprotic solvents with the *Z*-aminonitrone and the *E*-amino oxime as the minor forms. These theoretical studies were conducted on two compounds, benzamidoxime and acetamidoxime. *Z*-aminotrone and *E*-amidoxime are the most energetically stable after the dominant form *Z*-amidoxime: for acetamidoxime, the relative energies of these two tautomers are of 3.0 kcal/mol and 3.5 kcal/mol, respectively, while for benzamidoxime they are of 4.5 kcal/mol and 5.4 kcal/mol, respectively. The other tautomers, *E*-aminonitrone and all iminohydroxylamine isomers are less energetically favorable since their relative energies were higher than 8.5 kcal/mol in the case of acetoxime and of 9.8 kcal/mol for benzamidoxime. The nitroso-amine is always the less energetically stable isomer (ΔG ≈ 30 kcal/mol). The other tautomers show stabilities in between the tautomers mentioned above, the iminohydroxylamine exhibiting an intermediate stability with ΔG ≈ 10–13 kcal/mol (Figure 4) [16,18].

**Figure 5:**
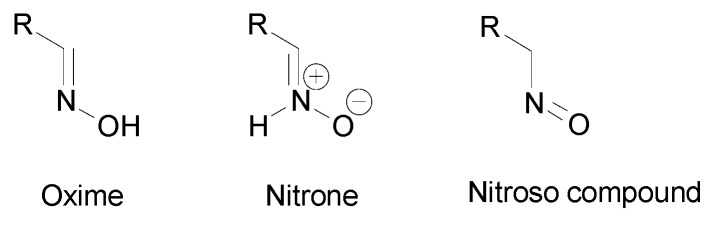
Tautomers of oximes.

Oximes tautomers were also studied and three main tautomeric forms were identified, the oxime, the nitrone and the nitroso compound (Figure 5) [19,20]. Depending on the structure, other cyclic tautomers may also exist [19]. These various isomers may present different biological activities [20] but no data can be found in the literature. The first identified isomerization was the oxime–nitrone [21]. Even if both nitrone and nitroso tautomers were identified to be less stable than the oxime form [22], the nitrone form exhibits a higher reactivity than the oxime, especially in cyclooaddition reactions and nucleophilic addition to unsaturated electrophiles [16,23]. The presence of an electron donating group on the oxime moiety allows to decrease the energy gap between the oxime and the nitrone form and stabilizes this latter, which facilitates addition reactions [16]. However, the oxime form is more reactive than the nitrone at high pH [24].

The oxime–nitrone tautomerism is the mostly studied in the literature and its mechanism was investigated. Recently, Lopez et al. demonstrated by theoretical calculations that this isomerization occurs via a bimolecular mechanism involving two oximes or two nitrones [23]. Previous studies suggested that the oxime–nitrone tautomerism likely originated from a thermal 1,2-hydrogen shift and a solvation effect [25,26,27,28].

Oximes can also exist as stereoisomers (*Z/E* forms). Numerous studies showed that the (*Z*)-oxime is the most stable configuration. This was proven by differential scanning calorimetry (DSC) studies in which heating and melting of the *E*-oxime afforded the *Z*-oxime. The oxime in its (*E*) configuration may be present with the *Z*-oxime but it represents the minor specie [20,25].

## 3. Synthesis of Oximes and Amidoximes

According to the literature, amidoximes and oximes are compounds easily synthesized in high yields. Amidoximes are usually prepared from the corresponding nitriles and hydroxylamine while oximes are generated from the aldehydes/ketones and hydroxylamine.

### 3.1. Synthesis of Amidoximes

Amidoximes are compounds possessing an amino and a hydroxymino function on the same carbon (Figure 1) [7]. The first synthesized amidoxime is formamidoxime obtained in 1873 by Lossen and Schigerdecker [29,30] but the first chemical structure of amidoximes only appeared in 1884 with Tiemann’s work [30,31]. Many synthesis methods of amidoximes were explored but the most commonly used nowadays is the nucleophilic attack of hydroxylamine on a nitrile (vide infra). Table 2, Table 3 and Table 4 summarize all the processes developed for amidoximes synthesis and reactions are classified by the type of additives used [7,30]. The methods described in Table 3 and Table 4 are scarcely used nowadays because they require more sophisticated procedures.

Table 2 shows that amidoximes can be prepared from hydroxylamine and nitrile, thioamide, amidine hydrochloride, hydrazide imide, iminoether, imidoylbenzotriazole, and pyrazine derivatives. The attack of hydroxylamine on nitriles is the mostly used method since Tiemann showed that mixing a nitrile with hydroxylamine hydrochloride and sodium carbonate in an alcohol produces the corresponding amidoxime after several hours of heating at 60–80 °C [31]. Via this method, amidoximes are generally prepared in high yield, up to 98% (entry 1) [32]. Synthetic methods described in entries 2 and 3 are more scarcely used since the starting compounds are prepared from nitriles [30]. In some cases, better results are obtained using thioamides than the nitriles [33]. The reaction of hydroxylamine with thioamides [30,33,34], amidines, hydrazide imides [30] or iminoethers [35], even if rarely used, usually affords the targeted amidoximes in yields varying from 60% to 100% (entries 2–4).

The use of the nitriles and hydroxylamine allows usually to obtain the expected amidoximes in good yields in a reaction time varying from of 1 to 48 h depending on the substrates. It is the leading method to prepare amidoximes using readily available nitriles as starting substrates [36,37,38]. Hydroxylamine hydrochloride is associated to bases like triethylamine or sodium carbonate (2 to 6 equivalents) that allow the in situ generation of hydroxylamine. The addition of hydroxylamine can be conducted at room temperature but is usually performed in refluxing ethanol or methanol to decrease the reaction time [36,37]. Aromatic amidoximes are obtained in higher yields than aliphatic ones. Nevertheless, it is still possible to improve the yield by adding an excess of hydroxylamine.

The use of an aqueous solution of hydroxylamine has also been reported. In this case, a base is not required and the reaction time is generally shorter than when using hydroxylamine hydrochloride [38,39]. This method has been demonstrated to be of interest for the efficient preparation of amidoximes starting from aliphatic nitriles.

Recently, Ranjbar-Karimi et al. synthesized a series of amidoximes using hydroxylamine and nitriles via a solvent free method under ultrasonic irradiation. The reaction proceeds in short time and allows the production of amidoximes in high yields (70–85%) [40].

Finally, the ring-opening of heterocycles using hydroxylamine was also demonstrated to be of interest for the preparation of amidoximes. One of the most recent methods was developed by Katritzky et al. by using imidoylbenzotriazoles and hydroxylamine as starting reagents under microwave irradiation (Table 2, entry 5). This method allows the fast preparation of amidoximes (reaction time of 5–15 min) and in good yields (65–81%) [41]. Hydroxylamine was also used for the synthesis of pyrazine-based amidoximes from open chain pyrazine derivatives or their pyrrolopyrazine tautomers. The reaction was demonstrated to occur either via the attack of hydroxylamine on the nitrile or the pyrrolidine ring. These reactions gave amidoximes in yields ranging from 63–93% after 18 h (Table 2, entry 6) [42,43].

The second synthetic process allowing the preparation of amidoximes involves the reaction of ammonia or amines with oximinoethers, hydroximic acids or nitric oxides (Table 3) [7,30]. The first example described in the literature is the reaction of ammonia with ethyl benzhydroximic acid for 8 h at 175 °C (Table 3, entry 1) [44]. This method is scarcely used but is of interest when using primary or secondary amines [30]. The use of 2 equivalents of amine relative to the hydroximic acid causes a fast dehydrohalogenation followed by a nucleophilic attack of the amine on the intermediate nitrile oxide (Table 3, entry 2) [30].

Some examples of reactions rarely described in the literature but allowing the preparation of amidoximes are described in Table 4. The reduction of benzonitrosolic acid with hydrogen sulfide was reported in 1906 by Wieland and Bauer (entry 1) [45].

Some reducing agents are able to open nitrogen containing heterocycles like 1,2,4-oxadiazoles, Δ′-1,2,4-oxadiazoles, 1-alkoxyadenines and 1,2,5-oxadiazoles [7,30]. The reaction of lithium aluminum hydride (LiAlH_4_) with Δ′-1,2,4-oxadiazoles allows the break of the C-O bond to give N-substituted amidoximes in 70% to 72% yield (entry 2) [46].

Finally, 1,2,4-oxadiazoles can also be transformed into amidoximes (yield of 75–85%) by reaction with aqueous NaOH [47]. Amidoximes were obtained in lower yields (35–63%) after passing 1-alkoxyadenine derivatives through Amberlite IRA 402 column and heating the eluate at 30–40°C for 7–48 h [48].

### 3.2. Synthesis of Oximes

An oxime is a compound belonging to imines family (RR′C = NOH). If R is an alkyl or an aryl group, R′ may be a hydrogen atom (aldoxime) or an alkyl/aryl group (ketoxime) (Figure 1). These compounds are usually synthesized by the addition of hydroxylamine on an aldehyde or a ketone [25,49,50]. The preparation of oximes is extensively described in the literature. Some improvements were described to enhance the yields and decrease the by-products formation. In 1999, Hajipour et al. reported the oximation of aldehydes and ketones using hydroxylamine hydrochloride under microwave irradiation in solvent free conditions. The process was demonstrated to be of higher efficiency for the synthesis of aldoximes using hydroxylamine hydrochloride and silica gel (yields higher than 76% and reaction time of 4 min) [51]. A similar solvent free method was reported in 2002 using hydroxylamine hydrochloride and zinc oxide giving yield of 80-98% and reaction time varying between 5 and 15 min at 140–170°C [52].

Recently, Li et al. reported an efficient synthesis of oximes under sonication (yields ranging 51 to 99% and short reaction times). The reaction is conducted in ethanol in the presence of anhydrous sodium sulfate [53].

Oximes can also be synthesized by the oxidation of aliphatic amines using *m*-CPBA. This reaction gave very high yields (>90%) in 20 min and at room temperature [54].

## 4. Oxidation of Amidoximes/Oximes and NO Release

During many years, NO was considered to present no beneficial biological effects and to be even toxic due to its presence in polluted environments [55]. In the 1980s, it was observed that a molecule released from the endothelium is responsible of the vasodilators effects and it was later identified as NO. Since, numerous studies on NO showed many biological benefits including its important role in vasodilation and blood pressure reduction [56]. For this reason, amidoximes and oximes were studied for their capacity of NO release.

### 4.1. Oxidation by Chemicals and Biomimetics

**Figure 6:**
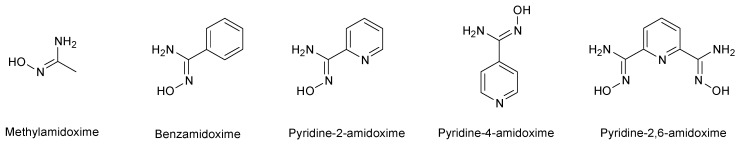
Amidoximes releasing NO in the presence of K_3_Fe[(CN)_6_].

Since the NO production in the human body originates from the oxidation of l-arginine by NOS, this oxidation route and the factors affecting the oxidation were investigated. In 1998, Koikov et al. studied the oxidation of three series of compounds including oximes and amidoximes at a concentration of 2 × 10^−4^ M with K_3_Fe[(CN)_6_] at pH 12 [57]. Under these conditions, oximes were not able of releasing NO and this phenomenon was explained by the acceptor or donor properties of the substituents (hydroxyphenyl and pyridine rings) and by the acidity of the oxime functions. However, many tested amidoximes showed the capacity to release NO in the presence of K_3_Fe[(CN)_6_] and methyl and phenyl amidoximes released 25% and 10% of NO, respectively. It was observed that replacing the phenyl ring by a pyridine ring allows to increase twice the amount of released NO and that the amidoximes exhibit quite a similar potential of NO release than methylamidoxime. The structures of these amidoximes are shown in Figure 6. The most important release was observed for the pyridine-2,6-diamidoxime for which the amount of released NO reached up to 40%. Benzonitrile was also identified as the major product present after the oxidation of benzamidoxime.

**Figure 7:**
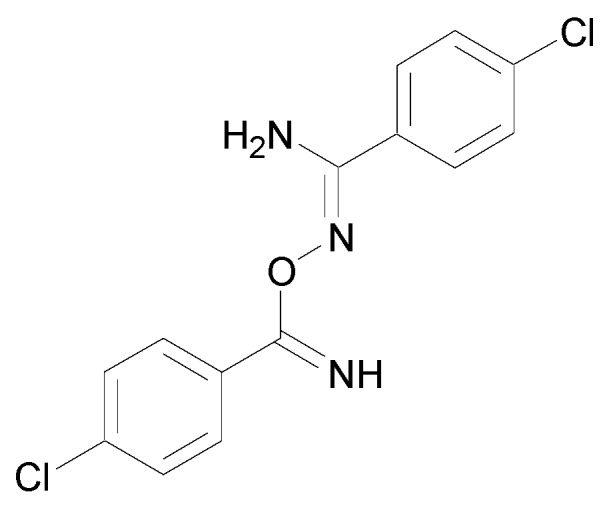
Dimeric product obtained from 4-chlorobenzamidoxime.

To better understand the products formed during the oxidation of amidoximes, Vadon-Le Goff et al. reported the use of various oxidants and biomimetic systems to study the reaction products as well as the causes and factors affecting the outcome of the reaction [4]. The authors evaluated the potential of NO release of 4-chlorobenzamidoxime in the presence of different oxidants. Amides or nitriles were identified as the main products but the presence of a dimeric product in the mixture was also detected (Figure 7).

It was demonstrated that with oxidants like Pb(OAc)_4_ and Ag_2_CO_3_, amidoximes are selectively oxidized into nitriles, while amides were selectively formed using oxidants capable of transferring one oxygen atom like H_2_O_2_, *t*-BuOOH or *m*-CPBA. Using *m*-CPBA, 4-chlorobenzamidoxime was oxidized into a mixture of dimeric products and amide, the latter being the major component. This oxidant, as well as *t*-BuOOH, associated with catalytic amounts of iron-porphyrin generates preferentially the nitrile in less than 1 h (ca. 80% of the amidoxime oxidized) (Table 5, lines 1–3). This likely originates from the formation of highly reactive iron-oxo species which oxidize the amidoxime into the nitrile. This result is in accordance with those described in the literature related to the oxidation of amidoximes by hemoproteins. Horseradish peroxidase, able of generating iron-oxo species in the presence of H_2_O_2_, oxidizes amidoximes to nitriles and a dimeric product. On the other hand, the oxidation by CYP450 generates exclusively the amide product instead of the nitrile. This was explained by the presence of the superoxide O_2_•^−^ radical anion or of a protein-metal-O_2_ complex instead of the presence of the iron-oxo intermediates (see Section 4.2 for the oxidation by CYP450).

**Figure 8:**
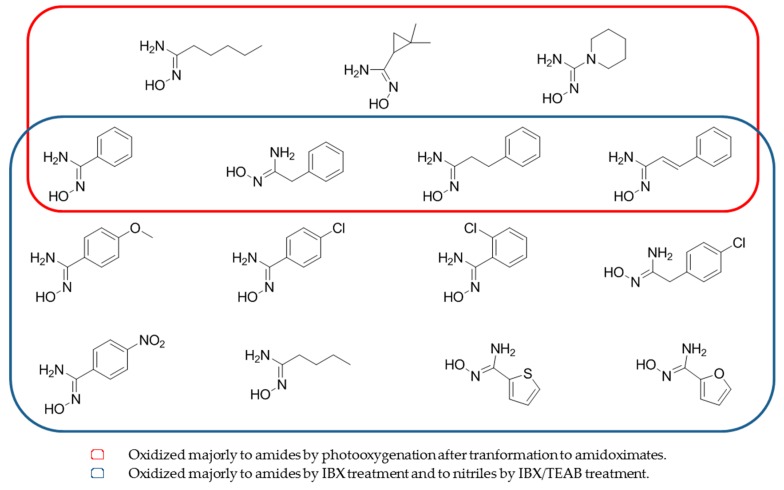
Structures of amidoximes able to be oxidized after photoxygenation or by treatment 2-iodobenzoic acid or IBX/tetraethylammonium bromide.

Amidoximes and oximes can also be oxidized via the amidoximate/oximate anions in the presence of singlet oxygen [58]. The authors demonstrate that amidoximes and oximes are inert to ^1^O_2_ but once converted to amidoximates and under photooxygenation, amidoximes can be oxidized into the corresponding amides along with nitriles as minor products (Table 5, entry 4). The use of ^1^O_2_ is similar to the oxidation of *N*-hydroxyguanidines by the NOS and clarifies the biological oxidation routes (Figure 8, red part).

To better understand the oxidation process of amidoximes, oxidants like 2-iodobenzoic acid (IBX) and the IBX/tetraethylammonium bromide (TEAB) combination were also used [12]. The authors demonstrate that IBX can selectively oxidize benzamidoxime (Figure 8, blue part) into amide (best yield of 83% using an amidoxime:IBX ratio of 1:1 and only 10% of the nitrile). This method was extended to various amidoximes and similar results were obtained. Nevertheless, the use of an amidoxime:IBX:TEAB molar ratio of 1:2:2 gave 90% of the nitrile and only 5% of the amide. By using this ratio with a series of amidoximes, similar results were obtained by the authors (Table 5, entries 5 and 6).

### 4.2. Biological Pathways of Amidoximes and Oximes Oxidation

As it is well known, NO is synthesized in vivo by the NOS enzymes. In recent years, many scientists got interested in finding new ways to oxidize amidoximes and oximes in order to increase the NO concentration in the organism. A few studies demonstrated that C=N-OH moieties present in compounds like amidoximes and oximes can be oxidized by CYP450 enzymes [59,60]. In 1998, a study showed the key role of CYP450 in the oxidation of amidoximes, oximes and *N*-hydroxyguanidines along with their oxidation products [5]. In the presence of rat liver microsomes, NADPH and O_2_, amidoximes and oximes can be oxidized and release NO and NO-related aerobic products like NO_2_^–^ and NO_3_^−^. The presence of both NADPH and O_2_ and of the active microsomes was demonstrated to be essential for the oxidation, indicating that this reaction is enzymatic. Moreover, the authors demonstrated that the CYP450 present in the microsomes are responsible for this reaction by using either the CYP450 inhibitor Miconazole which caused the inhibition by ca. 80–90% of the reaction or Dexomethasone (DEX), a CYP450 inducer, treated microsomes. The oxidation of various amidoximes and oximes generates both NO_2_^−^ and NO_3_^−^, especially in the presence of DEX-treated microsomes which boosts the oxidation. This release was always accompanied by the formation of amides and nitriles in the case of amidoximes and of ketones and nitroalkanes in the case of ketoximes.

In 2004, another study used CYP450 to test the oxidation of the oximes. Hence, Mäntylä et al. incubated buparvaquone–oxime and its derivatives with NADPH and either untreated rat liver microsomes or treated ones with various CYP450 inducers. These buparvaquone’s prodrugs were studied for their ability to be oxidized and releasing the corresponding buparvaquone alongside NO which was quantified via the NO_2_^-^ species produced [61]. All of the oximes were able to be oxidized in the presence of CYP450 and release buparvaquone bearing a C=O function effective against leishmaniasis. The presence of NO was also believed to have a role against this disease. The solubility of the oximes prodrugs was further improved by the synthesis of their corresponding phosphate prodrugs. These latter were able to release the corresponding oximes-buparvaquone [62].

**Figure 9:**
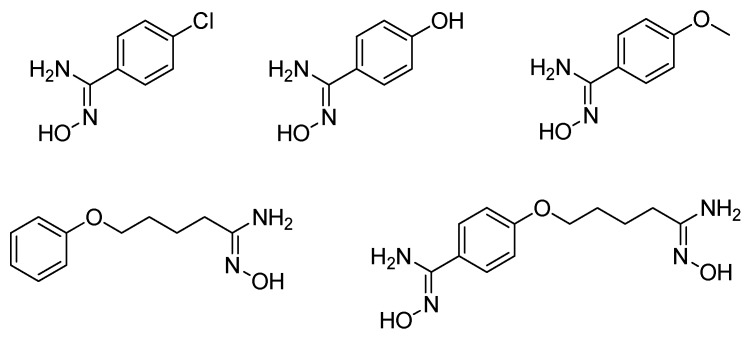
Structures of tested mono- and bis-amidoximes for NO release on rat liver microsomes and human vascular smooth muscle cells.

Recently, we evaluated the capacity of mono- and bis-amidoximes to release NO by a CYP450-mediated oxidation (Figure 9) [38]. Amidoximes were also incubated with NADPH and untreated microsomes. Only aromatic mono-amidoximes showed an important NO release while the aliphatic mono-amidoxime and the bis-amidoxime bearing both an aromatic and aliphatic amidoxime released only small NO quantities. The inadequate size or shape of the latter compounds and/or the lower reactivity of the aliphatic amidoximes compared to aromatic ones hinders their metabolization by rat liver microsomes. It was of interest to determine if these amidoximes are able to release NO once incubated with human cells not originating from the liver. For that purpose, these compounds were first demonstrated to be cytocompatible with human vascular smooth muscle cells (HVSMC) and were later incubated with these cells for 1 h to evaluate their ability to release NO. This test showed that all of the amidoximes are able to release NO and to increase the NO storage through nitrosothiol (RSNO) formation inside the cells. It is worth mentioning that the bis-amidoxime exhibited a high potential for the generation of RSNO, very close to that of the aromatic mono-amidoximes, but at a twice lower concentration.

Other reports focused on finding the capacity of the NOS in oxidizing amidoximes and oximes. Acetoxime can be oxidized by CYP450 and NADPH in the presence of metal complexes into NO_2_^–^ and reactive NO species [63]. However, this study clearly proved that acetoxime cannot be oxidized by the NOS II enzyme and cannot even inhibit its activity, which suggests a low affinity between NOS and this ketoxime. This demonstrates that the oxidation pathway of acetoxime in the cells involves only CYP450. In another study, four amidoximes were incubated with NOS I and II and NADPH and the NO formation was monitored by the Griess assay in order to study the amidoximes recognition by NOS [64]. All the amidoximes were inactive in the presence of NOS and did not show any NO release. Moreover, they are also very bad inhibitors of NOS I and II. The corresponding *N*-hydroxyguanidines analogs showed high affinity, indicating that the NOS better recognizes molecules bearing the –NH-C(R)=NH moiety than C=NOH. The latest study related to that topic was conducted in 2002 [65], and a series of *N*-hydroxyguanidines, amidoximes, ketoximes and aldoximes where tested for their NO release capacity in the presence of recombinant NOS II. The presence of an unmodified *N*-hydroxyguanidine function is mandatory for the oxidation by the NOS. Thus, none of the amidoximes and oximes could be oxidized by this enzyme.

As shown by these studies, CYP450 seems to be responsible of the oxidation of both amidoximes and oximes while NOS does not appear to have a role in these oxidations. As mentioned above, the oxidations by CYP450 afforded as major products amides in the case of amidoximes and ketones in the case of oximes alongside with the release of NO, nitrites and nitrates [63,64,65]. The mechanism of amidoximes and oximes oxidation by CYP450 that generates amides/ketones is explained by the formation of the superoxide radical anion (O_2_•^−^) which is a dissociation product of the CYP450-Fe(II)-O_2_ complex, this latter was shown to be easily transformed to CYP420-Fe(II)-O_2_ during the oxidation [60,63,64,65]. The formation, even in small amounts, of a nitrile and nitroalkane mixture originates from the presence of an iron-oxo complex as it is the case for the biomimetic reactions generating nitriles. Many CYP450 isoforms have been identified as responsible of the oxidations of these products like CYP4501A_1_, CYP4502B_1_, and CYP4502E_1_ [5,63].

These results demonstrate that NOS are not optimal for the evaluation of the NO release from amidoximes. It is preferable to use the CYP450 pathway that shows good results with both amidoxime and oxime functions.

### 4.3. In Vivo and In Vitro Biological Responses to NO Release from Amidoximes/Oximes Oxidation

Along with the discovery of the capacity of amidoximes and oximes to release NO in the presence of biological extracts and cells, many scientists started to test these products on biological tissues. Indeed, the in vivo generation of NO has gained a lot of attention among others due its capacity of vasodilatation and thus reducing platelet aggregation and thrombosis formation.

**Figure 10:**
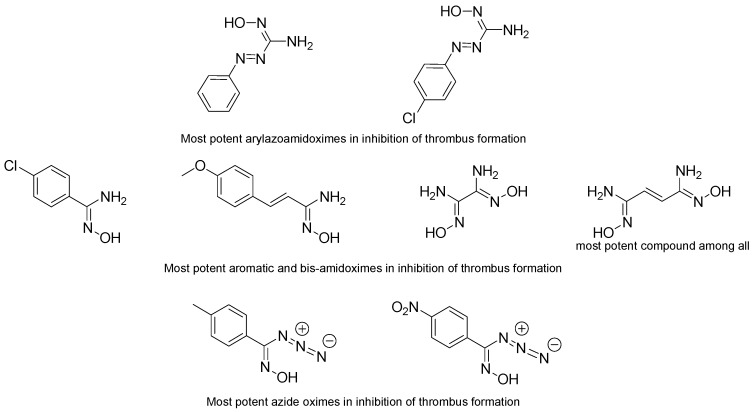
Most potent amidoximes and oximes in the inhibition of thrombus formation according to the studies of Rehse et al.

The first major works that appeared on that topic were published by Rehse et al. in 1997 and 1998 [66,67]. Seven aryl azoamidoximes and their effects on thrombosis inhibition, blood pressure and inhibition of platelet aggregation were investigated [66]. Five of the seven amidoximes inhibited the formation of thrombus in mesenteric rat vessels and even two of these amidoximes inhibited the thrombus formation by more than 20% (Figure 10). The remaining two amidoximes bearing a methoxy or a fluoro group on the *para* position did not show any significant effect on the thrombus formation in both arterioles and venules. The inactive 4-methoxyphenylazo-methanamidoxime had also no significant action in lowering the blood pressure during 2, 4, 6, and 24 h. The highly active 4-chlorophenylazo-methanamidoxime decreased the blood pressure for 24 h with a maximum efficiency of 16% ± 10% at 6 h. It is noteworthy that both amidoximes were able to release NO in the presence of DEX-treated rat liver microsomes and NADPH, but only the second amidoxime was used by the cells in order to lower blood pressure and reduce thrombi formation. The last test conducted with these amidoximes was the platelet aggregation. All of the studied amidoximes exhibited low or poor activity after calculating their IC_50_ following the Born test. This means that these amidoximes are not able of being oxidized in these conditions.

In another series of experiments, 17 compounds containing different aliphatic, aromatic, and bis-amidoximes were tested [67]. The results were in accordance with the previous study since the majority of the amidoximes showed a poor effect or even a lack of activity towards the inhibition of platelet aggregation which proves once more that the platelet rich plasma is not convenient for amidoximes oxidation. The most effective amidoxime was the aromatic one bearing a chlorine atom on the para position and an ethene group between the phenyl and the amidoxime function. The presence of this ethene group raised the efficiency for the platelet aggregation inhibition. Bis-amidoximes displayed a good activity for the inhibition of thrombus formation in arterioles and venules and the bis-amidoxime bearing an ethene group exhibited the highest percentage of thrombus inhibition. All other amidoximes also show some activity and it was noticed that adding a group on the para position may increase the thrombi formation inhibition, the key parameter being the lipophilicity rather than the electronic variations. The amidoximes exhibiting the highest activity in reducing the thrombus formation were also tested for decreasing the blood pressure but their activity was weak (the highest effect was observed for the bis-amidoxime with a blood pressure lowering of only 5%) (Figure 10).

These studies were not limited to amidoximes but also to azide oximes. These compounds showed better antiplatelet effect than the azide amidoximes and the amidoximes since the majority of the studied azide oximes exhibited a capacity to inhibit platelet aggregation with the oxime bearing the nitrophenyl function having an IC_50_ of 2 µmol/L in the Born test [68]. Similarly to amidoximes, the electronic variance and the lipophilicity had no major role in changing the efficiency of the oximes, however strong electron withdrawing groups like a nitro function seem to increase the oxime activity. All of the oximes showed also 10–20% efficiency in thrombus formation inhibition and two of them exhibited values higher than 20%. Additionally, azide oximes allowed a decrease of the blood pressure that may reach 10–15%. This parameter is not connected to the thrombus formation inhibition since the compound exhibiting the highest antithrombotic activity did not have the highest capacity of blood pressure lowering. Overall, the azide oximes showed to be more effective than the azide amidoximes and other studied amidoximes (Figure 10).

In another study and in order to evaluate the relaxation capacity of amidoximes, Jia et al. used formamidoxime in the presence of a tracheal ring previously contracted by carbachol, a cholinomimetic drug. They observed a relaxation effect on the tracheal ring after the incubation along with an accumulation of the cyclic guanosine 3′,5′-monophosphate (cGMP) level after incubation with the tracheal smooth muscle cells [69]. The relation between the production of cGMP and the relaxation of the ring was proved by using a cGMP inhibitor on both the tracheal rings and the smooth muscle cells. This inhibitor prevented the relaxation of the rings and the formation of the cGMP in both cultures. After the detection of NO in the incubation media of the tracheal smooth muscle cells with formamidoxime, the authors proposed that after the amidoxime oxidation, the released NO activates the formation of the cGMP which induces the relaxation of the tracheal ring. As previously, a NOS inhibitor was used with the cultured cells and the tracheal rings but it did not inhibit the production of cGMP or the relaxation of the rings. On the contrary, the cGMP production and the ring relaxation were both inhibited after the use of a CYP450 inhibitor, 7-ethoxyresorufin. Similarly, the cGMP production was inhibited after the use of Miconazole on the cell cultures. These results provided evidence that the pathway of the amidoxime oxidation in the tracheal smooth muscle cells and tracheal ring is through CYP450 and not NOS, and more specifically by CYP4501A_1_ being a strong substrate of the 7-ethoxyresorufin.

**Figure 11:**
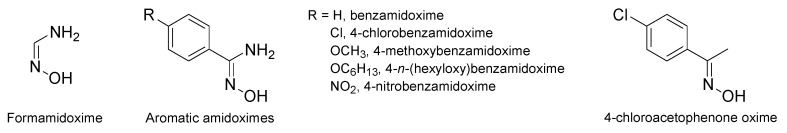
Some of the studied amidoximes and oximes for their activity in rat aorta relaxation.

This study using formamidoxime got a lot of attention and other researches were conducted to see if this compound, along with other amidoximes and oximes, exhibited the same effects in the rat aorta. Vetrovsky et al. tested a series of amidoximes and one oxime in the presence of endothelium-denuded rat aorta (Figure 11) [70]. All of the compounds caused an endothelium-independent relaxation higher than the NOHA with 4-chlorobenzamidoxime being the most active. It should also be noted that formamidoxime (Table 6, entry 1, in vitro studies) caused an important rat aorta relaxation close to that of the 4-chlorobenzamidoxime. The authors demonstrate that the presence of electron donating or withdrawing groups has a limited influence on the aorta relaxation since benzamidoxime, 4-methoxybenzamidoxime, and 4-*n*-(hexyloxy)benzamidoxime were slightly less potent than 4-chlorobenzamidoxime and 4-nitrobenzamidoxime. Additionally, the lipophilicity increase in 4-*n*-(hexyloxy)benzamidoxime compared to 4-methoxybenzamidoxime did not play a key role on the relaxation effect. Finally, the use of an oxime instead of an amidoxime function caused a decrease in the relaxation capacity.

Using the same tests but focusing on oximes like acetaldoxime, acetoxime and formaldoxime (Table 6, entries 2–4, in vitro studies), it was shown that all of these oximes are more potent to induce relaxation in rat aorta than hydroxyguanidine, formaldoxime being the most potent [71]. Similar to amidoximes, the lipophilicity increase from acetaldoxime to acetoxime does not significantly affect the induced relaxation. For the previously studied amidoximes and oximes, cGMP and NO were involved in the aorta relaxation since the addition of the guanylate cyclase inhibitor and NO-scavengers completely inhibited the relaxation. The use of NOS inhibitors and CYP450 inhibitors like proadifen did not alter the relaxation effect, indicating that other oxidation pathways are involved with these amidoximes and oximes. However, the use of the 7-ethoxyresorufin inhibited the relaxation of the aorta rings in the presence of 4-chlorobenzamidoxime and oximes but the authors suggested that this is not due to an inhibition of the CYP4501A_1_ but to an inhibition of a different NADPH-dependent reductase pathway. In a following study [6], the aliphatic oximes and formamidoxime (Table 6) were tested in vivo to evaluate the blood pressure decrease in conscious chronically cannulated rats in which the endogenous NO synthesis was blocked. In the precedent in vitro studies, the three tested oximes and the formamidoxime caused an aorta relaxation, contrariwise, in vivo only formamidoxime and formaldoxime were capable of lowering the blood pressure, with the amidoxime being the most active. This result was different from the in vitro experiments where the formaldoxime was the most potent. The inhibition of the guanylate cyclase in vivo caused the inhibition of the blood pressure reduction when using formaldoxime, which indicates that NO is responsible for the whole effect while with formamidoxime NO is responsible for one third of the effect, suggesting that with the amidoxime not the whole blood pressure decrease is caused by the NO release. Finally, hydrophilic substances were found to be more active in vivo than in vitro.

More recently, other studies evaluated the potency of some oximes to induce a vasorelaxation in superior mesenteric arteries isolated from rats. It was demonstrated that *E*-cinnamaldehyde oxime was the most potent and able of causing a NOS and endothelium-independent vasorelaxation. The NO release and relaxation activity of this compound were only partially inhibited by 7-ethoxyresorufin, a CYP4501A_1_ specific inhibitor, but not by other CYP450 nonspecific inhibitor, which shows also that the oxime oxidation is catalysed by a NADPH-dependent reductase pathway in the superior mesenteric arteries just like the studies in rat aortic rings. The relaxation pathway is also caused by the activation of cGMP with a key role played by a type of K^+^ channel and the reduction of the Ca^2+^ influx [72]. The role of the K^+^ channels was demonstrated in another study published in 2014 after the incubation of an oxime with rat aortic rings in the presence of different channel blockers [73]. The relaxation was observed when the enzyme and the aortic rings were incubated without the channel blockers. Furthermore, when administered to conscious rats, the oxime caused the reduction of blood pressure. The aorta and superior mesenteric artery rings relaxations were similar in the presence and in the absence of the endothelium after the incubation with the oxime, indicating that NOS were not responsible of the oxidation. The relaxation, as for the previously studied compounds, is achieved by the release of NO and the activation of the cGMP pathway alongside the activation of the K^+^ channels.

**Figure 12:**
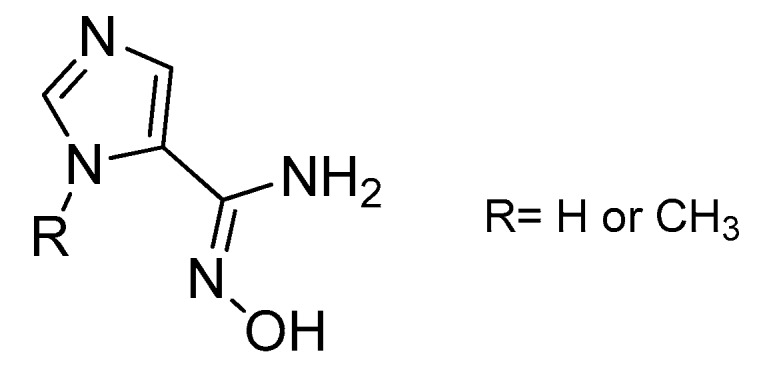
Imidazole amidoximes able to increase cGMP formation.

In addition, amidoximes can also be used for their capacity of lowering the intraocular pressure (IOP) in rabbits due to the NO release followed by the formation of cGMP [74,75]. In 2006, Oresmaa et al. tested various imidazole amidoximes for their IOP-lowering abilities. Two of them (Figure 12) were able to produce NO and increase the cGMP formation when incubated with porcine iris-ciliary bodies but only the methyl form was able to reduce the IOP when administered intravitreally in rabbits. The esterification of the active amidoximes caused the loss of their biological activity.

All of these studies demonstrate that when amidoximes or oximes are incubated with cultured cells such as tracheal smooth muscle cells [69] or with biological tissues such as aortic/tracheal rings [69,70], the generation of NO followed by the relaxation of the tissues is observed. This relaxation was proved to be caused by an increase in the cGMP accumulation after the activation of the guanylate cyclase by the released NO. Moreover, even in biological tissues, the NOS pathway does not seem to play a role in the oxidation of these products [69,70,71]. However, in some biological tissues like aortic rings and superior mesenteric arteries, CYP450 did not seem to play a role in the oxidation but it was suggested to be another NADPH-dependent reductase pathway [70,71,72]. Independently from the enzyme involved in the oxidation of amidoximes and oximes, it is now well established that the related NO release causes an activation of the guanylate cyclase with an accumulation of cGMP. This is followed by an activation of the BK_Ca_ channels which causes an efflux of the potassium ions and thus a decrease in the calcium channels activity [72,73]. This pathway causes a relaxation in the targeted tissue along the detected effects such as blood pressure decrease.

## 5. Conclusions

Amidoximes and oximes are compounds that are easy to synthesize and that present many benefits especially in biological pathways. In this review, we focused on their NO donor abilities. Many oximes and amidoximes can be oxidized by the CYP450 route rather than the NOS pathway which is of high interest when the patient’s NOS is not functional. Additionally, these compounds showed in vitro and in vivo abilities to cause the relaxation of aortas, to inhibit platelet aggregation and to decrease the hypertension. For all these reasons, amidoximes and oximes are of high potential for future use against many cardiovascular diseases.

## Figures and Tables

**Table 1:** Infrared bands in solid and liquid phases of amidoximes.

	Functions	Stretching Solid Phase (cm^−1^)	Stretching Liquid Phase ^a^ (cm^−1^)
Amidoxime form	OH	-	3620
	NH_2_	3400 and 3500 (sh)	
	NH_2_ scissor	1575–1620	
	Associated OH and NH_2_	2500 and 3300 (br)	
	C=N	1650–1670 (vs)	
Aminonitrone form	C=N^+^(H)	1690 (m – s)	-

^a^ liquid phase stretching is performed in chloroform; sh: sharp, br: broad, vs: very sharp, m: medium, s: strong.

**Table 2:** Amidoxime syntheses using hydroxylamine.

Entry	Hydroxylamine Action on	Reaction
1	nitrile	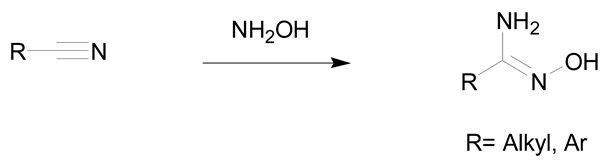
2	thioamide	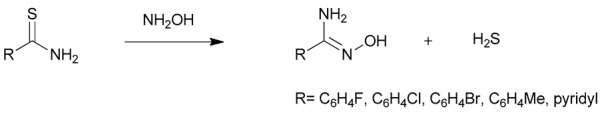
3	amidine hydrochloride or hydrazide imide	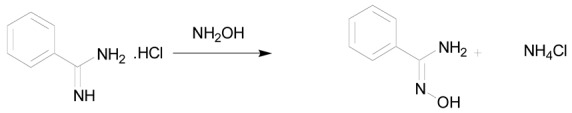
4	iminoether	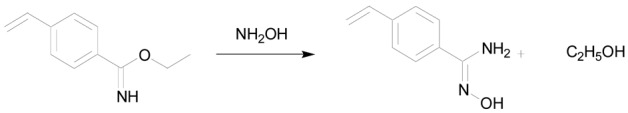
5	Imidoylbenzo-triazole	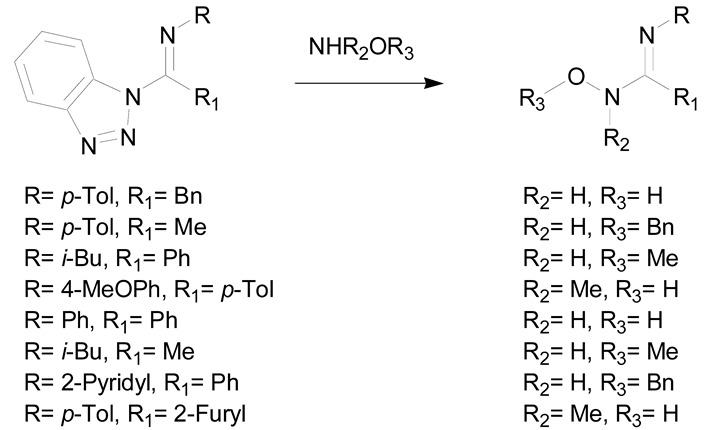
6	Open chain pyrazine derivatives and/or pyrrolopyrazine	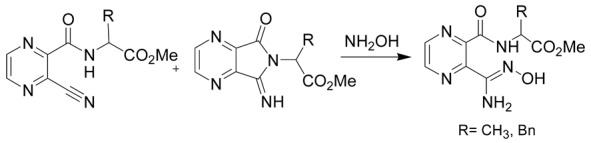

**Table 3:** Reaction of ammonia and amines with oximinoethers and hydroximic acid chlorides.

Entry	Ammonia and Amine Action on	Reaction
1	oximinoether	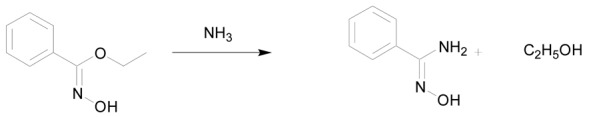
2	hydroximic acid chlorides	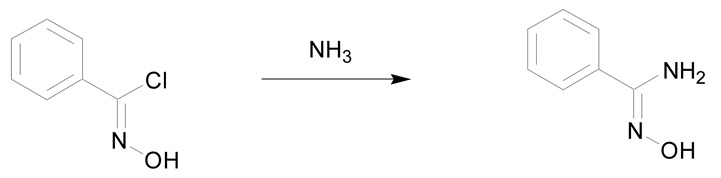

**Table 4:** Miscellaneous reactions allowing the preparation of amidoximes.

Entry	Method	Reaction
1	Reduction of nitrosolic acids and related compounds	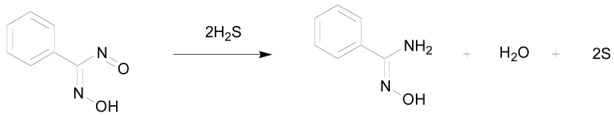
2	Ring opening of nitrogen-containing heterocycles	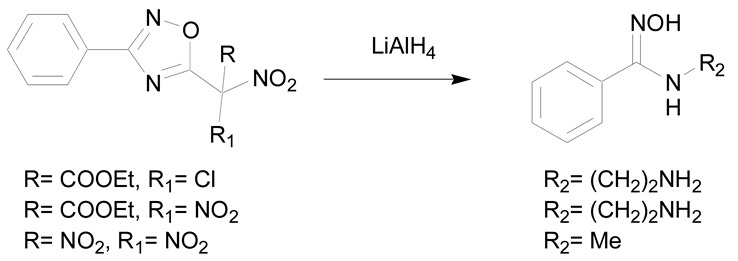

**Table 5:** Summary of the major oxidation products after amidoxime reaction with different chemical and biomimetic oxidants.

		Major Oxidation Products	
Entry		Amide	Nitrile
1	Monoelectronic oxidants		√
2	O_2_ atom donors	√	
3	O_2_ atom donors + iron-porphyrin catalyst		√
4	Photooxygenation of amidoximates	√	
5	IBX	√	
6	IBX/TEAB		√

√ Major product formed.

**Table 6:** Comparison of some studied amidoximes and oximes for their aorta relaxation and pressure decrease abilities.

Molecule	In Vitro Rat Aorta Relaxation	In Vivo Blood Pressure Decrease
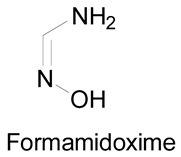	+++	+++
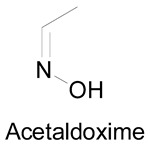	++	×
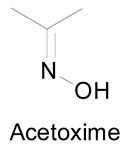	++	×
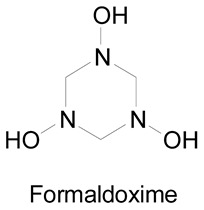	++++	++

++++ highly active; +++ active; ++ weakly active; × inactive.
